# The Use of Sodium Benzoate on Shelf-Life and Quality Attributes of Dried Chili Fish Paste Stored in Different Packaging Containers

**DOI:** 10.3390/foods10081802

**Published:** 2021-08-05

**Authors:** Jaksuma Pongsetkul, Soottawat Benjakul

**Affiliations:** 1 School of Animal Technology and Innovation, Institute of Agricultural Technology, Suranaree University of Technology, Nakhon Ratchasima 30000, Thailand; 2 International Center of Excellence in Seafood Science and Innovation, Faculty of Agro-Industry, Prince of Songkla University, Hat Yai 90110, Thailand; soottawat.b@psu.ac.th

**Keywords:** chili paste, sodium benzoate, packaging, shelf-life extension, storage

## Abstract

This study was carried out to assess the quality changes and shelf-life of dried chili fish paste treated with 0.1% sodium benzoate (SB) and stored in various packaging containers, including polypropylene (PP+SB), polyethylene-terephthalate (PET+SB), and LLDPE-aluminum Ziplock bag (ZL+SB) during 20-week storage at room temperature (25–28 °C) compared with samples without preservatives (PP, PET, and ZL). The result found that samples treated with 0.1% SB exhibited slower rate of quality changes throughout storage, including pH, browning index, oxidation products, as well as microorganisms, etc. These samples can store at room temperature for at least 20 weeks without any spoilage. Moreover, the sensorial scores of them, assessed by 50 untrained panelists who were familiar with this product, were more than 7.0 in all aspects, for example, color, flavor, and texture. In contrast, samples without preservatives, which revealed the higher rate of the changes in all quality characteristics, underwent spoilage during 20-week storage at different times depending on the packaging container. The shelf-life of PP, PET, and ZL were 6, 10, and 10 weeks, respectively, as indicated by the excess of total microorganisms (>1.00 × 10^4^ CFU/g sample). Overall, the results indicated that using sodium benzoate at the level of 0.1% can effectively extend the shelf-life of dried chili fish paste for at least 5 months with prime quality.

## 1. Introduction

“*Nham-prik*”, or chili paste, is embedded in Thai eating culture. Ninety-eight percent of Thai households consume certain chili pastes, while 64% of them consider the chili paste as their regular home dish [1]. There are many variations of chili paste across the country. Among all chili paste, “*Nham-prik-Narok-pla*”, or dried chili fish paste, is the most common type consumed by Thai people in all regions. The ingredients of dried chili fish paste generally include dried chili, garlic, shallot, palm sugar, tamarind juice, and grilled catfish. Grilled catfish added to this product is responsible for providing protein and some characteristic marine and dried seafood aromas. Rotsatchakul et al. [2] suggested that products from the Maillard reaction occurring during the heating process can create additional aroma components for chili-paste products. Normally, typical flavor and taste plays a profound role for good quality and consumers’ acceptance for this product. However, experienced consumers who consumed it regularly stated that the desirable flavor and odor as well as other quality characteristics change during storage period. Therefore, searching for packaging that does not only prolong the shelf life but also preserves the fresh/desirable characteristics of chili paste should be of importance.

Packaging is one of the most important factors impacting the shelf-life of the product. Good packaging should not only make food more attractive and convenient in use but also secures greater safety of the food against biological and chemical changes. Polypropylene-container (PP) and polyethylene-terephthalate (PET)-containers are the original packaging for dried chili fish paste used in Thailand due to its cheap, high availability and familiar use with chili-paste product. Recently, LLDPE-aluminum Ziplock bag (ZL) has been used as alternative packaging to improve consumers’ attraction since it seems to be more trendy and more comfortable to use. However, in terms of quality and shelf-life aspects, there was no previous research to evaluate these kinds of packaging containers precisely. Moreover, although there is a standard for chili-paste products in Thailand, the awareness of food safety is still limited. In general, chili-paste products are generally not labelled with a suggested shelf life and usually stored at room temperature until they are completely consumed. However, due to its ingredients and processing steps, dried chili fish paste contains a short-period shelf life (not more than 1 month at room temperature). Occurrence of mold on product’s surface is often observed if the packaging of the product or storage condition is not suitable. At this point, processors try to solve this problem by adding some preservatives to the product or searching for suitable packaging, which can preserve its prime quality as long as possible. Sodium benzoate (SB) (NaC_6_H_5_CO_2_) is one of the most popular preservatives that can be used in various food products. It is a natural preservative found in cranberries, prunes, cinnamon, cloves, and apples [3]. SB was announced as Generally Recognized As Safe, or GRAS, and the Food and Drug Administration (FDA) claimed that it can be use on foods. It has been generally reported to be used at concentrations below 0.1% [4]. SB has antimicrobial properties preventing the growth of bacteria and mold. Adding SB plays a role in prolonging the shelf life of fish and fish products effectively. These include catfish [3], sardine [5], smoked catfish [6], etc. For example, Pagarkar et al. [3] reported that SB at the level of 0.1–0.2% can extend the shelf life of catla fish steaks from 18 days (untreated) to 27 days without affecting the texture, taste, and appearance. However, there is no previous research studied on the effect of using SB with chili fish paste. Additionally, little information regarding the quality changes of chili fish paste stored in different packaging containers during storage exists. Therefore, this study aims to investigate the effect of different packaging containers (PP, PET, and ZL) on physical, chemical, microbiological changes as well as sensory acceptability of dried chili fish paste stored at room temperature (25–28 °C). The potential of using 0.1% SB on shelf-life extension of this product stored in different packaging containers was also evaluated.

## 2. Materials and Methods

### 2.1. Preparation of Dried Chili Fish Paste

The ingredients used for producing dried chili fish paste include (1) grilled catfish meat (*Clarias macrocephalus* × *Clarias gariepinus*) (38.0%), (2) red chili (cv. *Capsicum frutescense*) (7.5%), (3) garlic (cv. *Allium sativum*) (22.5%), (4) shallot (cv. *Allium ascalonicum*) (17.5%), (5) palm sugar (5.5%), (6) tamarind juice (3.0%), (7) salt (3.0%), and (8) sugar (3.0%). For chili-paste preparation, firstly, red chili, garlic, and shallot were roasted in a hot-air oven at 180 °C for 20 min. After that, dried vegetables were ground into uniformity using a blender (National, Tokyo, Japan) to make a fine paste. Ground vegetables were mixed together with grilled catfish meat and blended more for 1 min at high-speed level. Then, all seasoning ingredients, including palm sugar, tamarind juice, salt, and sugar were added to the mixture before frying with a pan for 5 min. Dried chili fish paste, after left to cool at room temperature for 15 min, was ready for further analysis.

### 2.2. Chemical and Microbiological Characteristics of Dried Chili Fish Paste

Dried chili fish paste was ground to uniformity using a blender before being subjected to analysis as follows:

#### 2.2.1. Physical and Chemical Analysis

Moisture, protein, fat, ash, carbohydrate, and salt content were determined according to AOAC methods [7]. The pH of sample was measured by homogenizing 10 g of sample with 100 mL of distilled water and measuring a homogenate using a digital pH meter (Sartorius, Gottingen, Germany). Water activity (A_w_) was determined after equilibration at room temperature (~25 °C) using a water activity analyzer (Thermoconstanter, Novasina, Switzerland). Color of the ground sample was measured using an automatic colorimeter (ColourFlex, Hunter Lab Reston, Reston, VA, USA), and the result was expressed as L* (lightness), a* (redness/greenness), and b* (yellowness/blueness) [8]. Inorganic contaminants, including Pb, As, Cd, and Hg, were measured using acid digestion and quantification by ICP-OES (inductively coupled plasma–optical emission spectrometry), as per the method of Morgano et al. [9]. Aflatoxin contaminations, including Aflatoxin B1, B2, G1, and G2, were detected using the HPLC (DIONEX, California Avenue, Palo Alto, CA 94304, USA), as described by Erkmen [10].

#### 2.2.2. Microbiological Analysis

For microbial safety and populations, total viable count (TVC), yeast and mold count, as well as some pathogenic bacteria, including *Salmonella* spp., *Staphylococcus aureus*, *Bacillus cereus*, *Clostridium perfringens* and *Escherichia coli*, were detected following the method of BAM [11] and Thai Community Product Standards [12]. Ground chili paste (25 g) was mixed with 225 mL of 0.1% peptone water using a Stomacher 400 Lab Blender (Seward Ltd., Worthing, UK) at high speed for 3 min. The samples, with appropriate serial tenfold dilution, were cultivated in each specific media (Xylose lysine desoxycholate (XLD) agar for *Salmonella* spp., Trypticase (triptic) soy agar (TSA) for *S. aureus*, mannitol egg yolk polymixin agar (MYP, Difco, (Darmstadt, Germany) for *B. cereus*, Tryptose-sulfite-cycloserine (TSC) agar for *C. perfringens*, as well as Levine’s eosin-methylene blue (L-EMB) agar for *E. Coli*) and incubated following the procedure of individuals. Then, the microbial populations were counted and reported as colony forming units/g sample (CFU/g sample).

#### 2.2.3. Sensory Evaluation

Sensory evaluation of dried chili fish paste was carried out by 50 untrained panelists who consumed chili paste regularly, using 9-point hedonic scale [13]. Sample was served in a white paper plate at room temperature. Evaluation was made in individual sensory evaluation booths under fluorescent white light. The scores (1 = dislike extremely, 9 = like extremely) given by the panelists for each of the attributes (appearance, color, flavor, texture, and overall likeness) were pooled, and the average scores are presented.

### 2.3. Effect of Different Packaging Materials Incorporation with Sodium Benzoate (SB) on Quality and Shelf Life of Dried Chili Fish Paste during Storage

Dried chili fish paste was divided into 2 groups, including (1) control (without adding SB) and (2) added SB at the concentration of 0.1% (*w*/*w*). Then, samples were packed in 3 different packaging containers (80% of total volume).

(1) Polypropylene (PP) container with red screw cap;
(2) Airtight, clear polyethylene-terephthalate (PET) container with aluminum easy-open lid; and
(3) Translucent LLDPE-aluminum Ziplock bag (ZL) (14 × 20 cm).

All samples were stored at room temperature (25–28 °C) for 20 weeks. During storage, samples were collected to monitor on their quality changes every 2 weeks as follows:

#### 2.3.1. Physical and Chemical Analysis

- Moisture content, pH, water activity (A_w_), and color (as described in Section 2.2.1)
- Browning index (A_420_)

The browning index of dried chili fish paste was measured according to Pongsetkul et al. [8]. The samples were diluted (1:25 (*w*/*v*)) with distilled water and homogenized at a speed of 10,000× *g* for 2 min using an IKA Labortechnik homogenizer (Selangor, Malaysia). The absorbance was read at 420 nm using UV-1601 spectrometer.

- Lipid oxidation products

Lipid oxidation products, including peroxide value (PV) and thiobarbituric acid reactive substances (TBARS) value, were measured following the method of Takeungwongtrakul and Benjakul [14]. For PV, a standard curve was prepared using cumene hydroperoxide at the concentration range of 0.5–2 ppm, and the PV was reported as mg hydropreoxide/kg dry weight sample. For TBARS value, a standard curve was prepared using malonaldehyde bis (dimethyl acetal) at concentrations ranging from 0 to 2 ppm. TBARS value was expressed as mg malonaldehyde (MDA)/kg dry weight sample.

#### 2.3.2. Microbial Analysis

TVC as well as yeast and mold count of samples during storage were monitored with the same method of 2.2.2 except all 5 pathogens, including *Salmonella* spp., *S. aureus*, *B. cereus*, *C. perfringens*, and *E. coli*, which were counted only on the 20th week of storage (final day). Moreover, the amount of inorganic contaminants and aflatoxins was measured on the final day of storage to confirm the food safety of the product.

#### 2.3.3. Sensory Evaluation

Sensory acceptability of dried chili paste during storage was measured. All 6 samples were collected every 4 weeks to assess their scores with the same method of 2.2.3 Samples were coded with three-digit, random numbers and randomly served. During sensory assessment, panelists were allowed to take a rest for 5 min every 3 samples. Panelists were also instructed to rinse their mouths with water and consume bread before moving to assess the next sample.

### 2.4. Statistical Analysis

All experiments were run in triplicate using three different lots of samples. Data were subjected to analysis of two-way analysis of variance (ANOVA) using the SPSS package version 11.0 (SPSS for window, SPSS Inc., Chicago, IL, USA). Significant means were compared at 5% probability level using the Duncan’s multiple range test (DMRT) following the method of Steel et al. [15].

## 3. Results and Discussion

### 3.1. Chemical Composition, Physical Properties, Microbial Population, and Sensory Evaluation of Dried Chili Fish Paste

#### 3.1.1. Physical and Chemical Characteristics

Proximate composition of dried chili paste is presented in Table 1. This chili fish paste is a good source of protein, as it is a major constituent at 37.63%. Lipid content was quite high (11.12%). This was due to the fact that the sample was made from grill catfish, accounting for 80% of its recipe. Fresh catfish, used as raw material, has a protein and fat content of 20.62% and 8.66%, respectively, as reported by Thanonkaew et al. [16]. During grilled fish preparation, the frying process can remove moisture from the fish and leads to a higher concentration of lipid content in fish muscle. Dried chili fish paste contained salt and ash content of 3.02% and 15.61%, respectively. Slightly high ash content was more likely attributed to the presence of fibers or inorganic substances of ingredients, especially spices and herbs, used in recipe, such as garlic, shallot, chili, etc. Moreover, the quite high content of carbohydrate (18.19%) was related to the adding of palm sugar and sugar during production to enhance its taste. Moisture content of dried chili fish paste was 17.45%. As per the standard of this product [12], the moisture content of chili paste is regulated so as to not have more than 20%. During production, drying process of each ingredient and frying all ingredients together were therefore important processes to remove excess water from the final product. Low moisture content was in agreement with the low water activity, or A_w_. The pH and A_w_ of dried chili fish paste was 5.72 and 0.72, respectively. Dried chili fish paste can be classified as an intermediate moisture food (IMF), with an A_w_ of about 0.7 [17]. Lowered A_w_ can retard the spoilage caused by chemical and microbiological reactions and thus could be associated with the prolonged shelf life of this product. However, with its A_w_, this chili paste may be susceptible to spoilage by mold due to its suitable condition for mold’s growth [18].

Lightness (L*), redness (a*), and yellowness (b*) of dried chili fish paste are 27.55, 14.49, and 26.50, respectively (Table 1). The product had low L* and a*-values but a dominantly high b*-value. This indicated that this chili paste is dark-yellow-brown in color. The yellowness and brownness were governed via non-enzymatic browning reactions, especially the Maillard reaction. Reducing sugar (from sugar/palm sugar) as well as the carbonyl groups of aldehydes and ketones, the oxidation products, could react with amino groups of free amino acids or peptides (from grilled catfish), leading to yellow or brown color development of this product [19].

Aflatoxins (AFs), including AFB1, B2, G1, and G2 type as well as inorganic substances, including Pb, As, Cd, and Hg were also examined to confirm the product’s safety. Normally, the molds in chili pepper are one of the highest contaminants, and mycotoxins, including aflatoxin, have been frequently detected in chili and other spices [20]. AFs, a kind of mycotoxins, are highly toxic secondary metabolic products of molds belonging to *Aspergillus* spp. and have carcinogenic, mutagenic, and teratogenic effect on humans and most animals [21]. The rank order of toxicity of AFs is AFB1 > AFG1 > AFB2 > AFG2, respectively. The allowed maximum limits of total aflatoxin for chili fish paste is not more than 20 μg/kg sample [12]. Undetectable levels of all types of AFs were found (Table 1). This indicates that there was no risk of AFs from raw materials, which can cause the contamination of the final product and effect on consumers’ health. There are some inorganic substances limited by TCPS due to the safety of the product. Those include Pb, As, Cd, and Hg, which should not more than 1, 2, 1, and 2 mg/kg sample, respectively [12]. The result also revealed that there was no Pb and As found in our dried chili fish paste. Moreover, the amount of Cd and Hg (<0.60 and 0.27 mg/kg sample) were in the range limited by Thai Community Product Standard (TCPS) [12]. Normally, toxins or inorganic substances always contaminate during improper processes, such as low quality of raw materials, poor hygiene, improper storage, etc. These results indicated that fresh chili fish paste is safe to consume.

#### 3.1.2. Microbial Population

Microbial population and pathogens of dried chili fish paste were evaluated following the Thai Community Product Standard [12] and depicted in Table 1. Sample had the amount of total viable count (TVC) of 4.58 × 10^2^ CFU/g sample. The amount of total microorganisms was in the range of the standard, which is regulated as not more than 10^4^ CFU/g sample [12]. Moreover, the yeast and mold count of the chili paste (<10 CFU/g sample) as well as the amount of all pathogens, including *Salmonella* spp., *S*. *aureus*, *B*. *cereus*, *C*. *perfringens*, and *E*. *coli*, were also not more than the limitation regulated by TCPS [12]. This microbial information in addition with the determination of AFs and inorganic substances, which are major hazards found in this product, revealed that this product is safe to consume. The absence of chemical hazards or toxins and other pathogenic bacteria could be attributed to the proper hygiene and good manufacturing practices during processing. However, the amount of all microorganisms, especially yeast and mold and also pathogens, can change during storage, affecting the safety and the shelf life of the product to some extent.

#### 3.1.3. Sensory Evaluation

Sensory evaluation of dried chili paste was carried out by 50 panelists using 9-point hedonic scale in relation to the product’s appearance, color, flavor, texture, and overall-liking scores (Table 1). The result showed that the sample had a score higher than 7 in all aspects, indicating the high acceptability of the product by consumers. However, it was normally noted that chili paste quality can be changed during storage, especially in flavor and odor. This led to lower consumers’ satisfaction. Therefore, packaging that can avoid or delay those quality changes as well as preserve its original quality are the most important things for searching/evaluating. Suitable packaging does not only extend the shelf-life of chili fish paste but also increases its marketable value.

### 3.2. Effect of Different Packaging Materials’ Incorporation with Sodium Benzoate (SB) on Quality and Shelf Life of Dried Chili Fish Paste during Storage

Monitoring on the quality changes of dried chili fish paste mixed with/without 0.1% SB and kept in different packaging containers during storage at room temperature were examined every 2 weeks. Overall, each sample had a different rate of physical, chemical, as well as microbiological changes, which led to the difference in the shelf life of the product. Among all quality characteristics, total microorganism as well as yeast and mold counts were the key to eliminate unaccepted product, following the standard of this product [12]; thus, each sample was judged as spoiled on different days of storage. For the safety aspect, only samples having a microbial population in the range regulated by Thai standard were examined on their sensory evaluation by panelists up to 20 weeks.

#### 3.2.1. Changes in Physical and Chemical Characteristics

##### Changes in Moisture Content, A_w_, pH, and Color

Moisture content of dried chili fish paste with/without 0.1% SB and stored in different packaging containers showed a slightly increasing trend (Table 2). Moisture content of 17.45% at day 0 of storage continually increased as storage time increased and reached 19.25–20.65% at the 20th week of storage. The increase in moisture content results in the increase in A_w_ with increasing storage time (data not shown). Normally, high moisture content or high water activity plays a role to limit the shelf life of dried or semi-dried products. The standard of chili paste in Thailand [12] regulates the moisture content of this product at not more than 20% (*w*/*w*); therefore, this parameter can be used for judging the shelf life of the product. Among all samples, PET had the highest rate of increasing moisture, and the moisture content reached 20% at 12th week of storage. Keller and Kouzes [22] reported that the water permeability of PET at room temperature was 130 cm^3^ × cm × cm^−2^ × S^−1^ × cm-Hg^−1^, which was higher than PP and LLDPE (material for zip-lock bag) (35 and 68 cm^3^ × cm × cm^−2^ × S^−1^ × cm-Hg^−1^, respectively). The higher water permeability of plastic/material of packaging favored moisture migration from the environment through the packaging, resulting in the increase in moisture content of the product. It was also noted that dried chili paste added with 0.1% SB showed lower rate of moisture increase during storage compared with the samples without added SB. This corresponded well with the lower rate of A_w_ of those samples as well. At the 20th week of storage, samples having 0.1% SB, including PP+SB, PET+SB, and ZL+SB, had a moisture content of 19.78%, 19.25%, and 19.87%, respectively, which was in the range of the limitation of the standard. However, the samples without 0.1% SB, including PP, PET, and ZL, had a moisture content over 20% within weeks 14, 12, and 20, respectively. This indicated that dried chili fish paste without added 0.1% SB had a shelf life lower than 20 weeks at room temperature due to the excess moisture content.

The pH of dried chili fish paste, which was 5.72 at day 0, gradually decreased as storage time increased (data not shown). The pH dropped to 4.91–5.39 when stored for 20 weeks. The rate of pH change was similar with the changes in other characteristics. Samples with added 0.1% SB had a slower rate in quality change compared with the samples without preservatives at all periods of storage time. The decrease in pH may relate to the increase in total microbial populations (Table 4). During storage, some microorganisms can use carbon sources, small peptides, or amino acids as nutrients for growth and can produce acid as products, such as lactic acid bacteria [23], resulting in the decrease in pH of the product. This phenomenon was also in agreement with the slower rate of pH decrease in samples without added preservatives. Normally, SB has antimicrobial properties preventing the growth of bacteria and mold [3]. The inhibition of some microorganisms by SB may delay the production of acid by those microorganisms to some extent.

**Figure 1:**
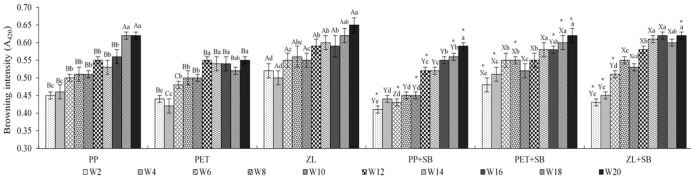
Change in browning intensity (A_420_) of dried chili fish paste during storage in various packaging at room temperature. Different uppercase letters indicate significant differences (*p* < 0.05) due to packaging material at the same period of storage time (without 0.1% SB: A,B,C; with 0.1% SB: X,Y,Z). Significant differences between with/without added 0.1% SB in samples stored in the same packaging material are indicated by an asterisk (*) on the added preservative samples (*p* < 0.05). Different lowercase letters indicate significant differences (*p* < 0.05) due to storage time.

Changes in color of dried chili fish paste stored in different packaging containers during storage at room temperature were observed (Table 3). L*-value of all samples slightly decreased as storage time increased (*p* < 0.05). The lightness in color decreased from 25.92–28.42 at day 0 to 19.63–24.08 at the 20th week of storage, indicating the significantly darker color of samples when the storage time was extended. The change in a* -value or redness was slightly fluctuant throughout storage; however, most samples had no differences in a* -value when stored for 20 weeks compared with day 0 (*p* > 0.05). This indicated that the red color of the product, which mainly come from dried chili (used as 7.5% of overall ingredients), was not changed during storage for 5 months. However, b*-value was in contrast. The b*-value or yellowness of dried chili fish paste was obviously increased as storage time increased (*p* < 0.05). The increase in b*-value as well as the decrease in L*-value revealed that the samples turned darker or browner in color. The decrease in moisture content during prolonged storage time may result in the darker or more intense color of the products. Change in color was also in agreement with the increase in browning index (Figure 1). Pomsa et al. [24] reported that the decrease in L* -value of roasted chili paste mixed with mung bean hull was observed when storage time increased, indicating the darker color of chili-paste product when the storage time was extended. Normally, color is one of the most important factors affecting consumers’ acceptability. However, change in color of all samples in this study did not affect sensory scores (in the aspects of color-liking score) during storage for 20 weeks, as shown in Table 5.

##### Changes in Browning Index (A420)

Figure 1 reported the change in browning index of dried chili fish paste during storage at room temperature. Generally, browning index indicated the browning development in the final stage of the Maillard reaction [25]. It was noted that the browning index of all chili-paste samples continually increased as storage time increased up to 20 weeks. The increase in browning index was in agreement with the changes in color (Table 3). This change was also associated with the increase in moisture content (Table 2) and water activity as well. The result indicated that extended storage time could favor Maillard browning reactions. Normally, water activity above 0.3 is known to cause non-enzymatic (Maillard) browning if the product is susceptible to such reactions [19]. The rate of nonenzymatic reaction increases with increasing water activity, reaching a maximum at water activity (A_w_) ranging from 0.6 to 0.75 [19]. During storage, A_w_ of all samples increased from 0.72 (day 0) to 0.79 (week 20), which is suitable for this reaction. Moreover, the ingredients used to produce chili paste, especially carbonyl group from sugar or palm sugar, as well as carbonyl groups from grilled catfish can be well served as substrates for the Maillard reaction. In addition, the high relative humidity of the storage environment was therefore assumed to have influenced the rate of browning reactions of samples stored in different type of packaging/plastic. A relationship between color development, especially changing to darker or browner, resulting from Maillard reaction products, pH, temperature, and packaging material, has been demonstrated by much previous research [19,21,26,27].

##### Changes in Lipid Oxidation Products

**Figure 2:**
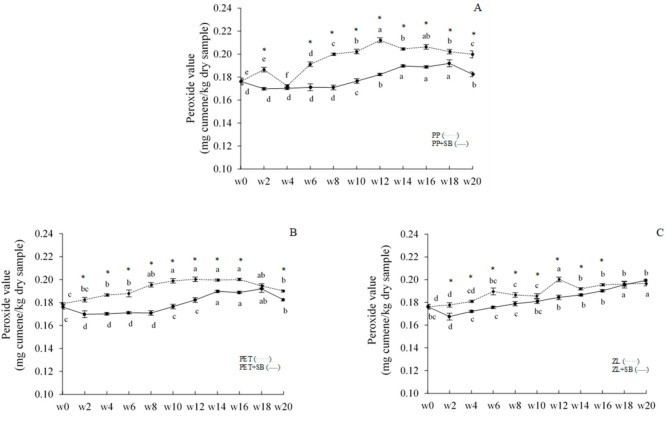
Change in peroxide value (PV) of dried chili fish paste during storage in various packaging at room temperature (25 °C). (**A**): PP (^. . . . . .^)/PP+SB: (^____^), (**B**): PET (^. . . . . .^)/PET+SB: (^____^), and (**C**): ZL (^. . . . . .^)/ZL+SB: (^____^). Significant differences between with/without added 0.1% SB in samples stored in the same packaging material are indicated by an asterisk (*) (*p* < 0.05). Different lowercase letters in the same column denote significant differences (*p* < 0.05) due to storage time.

**Figure 3:**
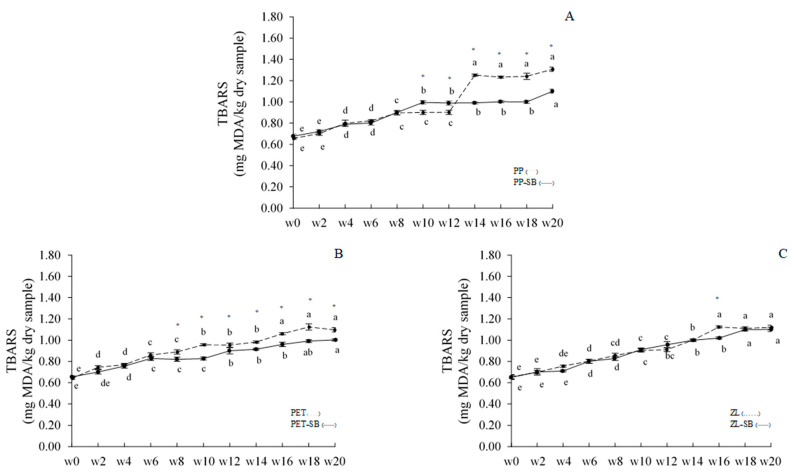
Change in TBARS value of dried chili fish paste during storage in various packaging at room temperature. (**A**): PP (^. . . . . .^)/PP+SB: (^____^), (**B**): PET (^. . . . . .^)/PET+SB: (^____^), and (**C**): ZL (^. . . . . .^)/ZL+SB: (^____^). Significant differences between with/without added 0.1% SB in samples stored in the same packaging material are indicated by an asterisk (*) (*p* < 0.05). Different lowercase letters in the same column denote significant differences (*p* < 0.05) due to storage time.

Lipid oxidation products of dried chili fish paste were monitored throughout 20 weeks of room-temperature storage (Figure 2 and Figure 3). At day 0, PV of chili paste was 0.1762 mg/kg sample. PV of all samples increased as storage time increased (*p* < 0.05), especially at the first period of storage. PV of most samples was constant or slightly decreased when extending the storage time except for the ZL+SB sample. PV of ZL+SB continually increased throughout 20 weeks and reached the maximum at 0.1994 mg cumene/kg sample. Normally, PV is a good indicator of lipid oxidation under normal conditions or room temperature [28]. Peroxides are the initial reaction products of lipid oxidation and responsible for primary oxidation. However, the hydroperoxide subsequently breaks down and forms secondary oxidation products during storage. The decrease in PV in later weeks was well in accordance with the increase in TBARS value (Figure 3). Dried chili fish paste had the TBARS value of 0.6502 mg MDA/kg sample at day 0 of storage. The increase of TBARS value as storage time increased was monitored in all samples (*p* < 0.05). TBARS value of all samples reached the maximum level at 20th week of storage (1.0024–1.3067 mg MDA/kg sample). Changes in PV and TBARS indicated well that lipid oxidation occurs in all samples during storage. These compounds may cause bad characteristics, especially flavor and odor of the product, which may relate to the decrease in flavor-liking scores when extending the storage time of samples tested by panelists (Table 5). It was also observed that the rate of the increase in PV and TBARS value was governed more by preservative added than by the packaging container. Compared between samples stored in the same packaging container, higher lipid oxidation products were mostly found in samples with added 0.1% SB throughout the storage (*p* < 0.05). At the end of storage, both PP+SB and PET+SB had significantly lower PV and TBARS than those without preservative. This indicated that adding 0.1% SB can retard lipid oxidation damages effectively, which may be associated with the limitation of some bacterial growth, especially lipase-producing bacteria, etc.

#### 3.2.2. Changes in Microbial Population

Total viable count (TVC) as well as yeast and mold count of dried chili fish paste stored in various packaging containers with/without 0.1% SB are shown in Table 4. TVC of fresh sample (day 0) was 4.58 × 10^2^ CFU/g sample. The continuously increase in TVC during storage were observed in all samples (*p* < 0.05). However, each sample contained different rates of microorganisms changing. It was clearly noted that samples with 0.1% SB can maintain the amount of TVC greater than samples without added SB. TVC of PP+SB, PET+SB, and ZL+SB slightly increased throughout storage up to 5 weeks. At the end of storage, the amount of TVC was lower than 1.00 × 10^4^ CFU/g sample, which is still in the range regulated by microbial standard of this product [12]. Moreover, TVC of these three samples were not much different at all periods of storage (not more than 1 log CFU). This indicated the effectiveness of SB (at 0.1%) for inhibition of some microorganism growth during storage. Moreover, the result also revealed that TVC can be used as an index to determine the shelf life of this product. TVC of samples without added 0.1% SB increased rapidly during storage and were over the limit within 6 weeks for PP sample (1.05 × 10^4^ CFU/g sample) and 10 weeks for PET and ZL (2.98 × 10^4^ and 1.22 × 10^4^ CFU/g sample, respectively). Therefore, dried chili paste without preservatives had a shelf life lower than 10 weeks, while adding 0.1% SB can extend the shelf life of this product for at least 20 weeks at room temperature.

The increase in yeast and mold count was observed as storage time increased, a similar trend with the increase in TVC (Table 4). Among all samples, PP exhibited the greater increase in yeast and mold count compared with others (*p* < 0.05), while dried chili fish paste with added 0.1% SB (PP+SB, PET+SB, and ZL+SB) had a slower rate of increase throughout storage. Based on the standard of this product regulated by TCPS [12], chili paste should have yeast and mold count not more than 100 CFU/g sample. The result was corresponding with the amount of total microorganisms during storage in each sample. Samples having 0.1% SB had yeast and mold counts lower than 100 CFU/g sample throughout storage time of 20 weeks. In contrast, yeast and mold count of PP was 106 CFU/g sample at 6 weeks of storage, indicating the limitation of shelf life itself at this week. In contrast, yeast and mold counts of PET and ZL were exceeded at 12 and 14 weeks, respectively (102 and 115 CFU/g sample). However, the shelf life of two latter samples was limited at 10 weeks (as indicated by the exceed of TVC). Normally, yeast and mold can grow well at intermediate moisture food (IMF) or product having the A_w_ of 0.7–0.85 [6]. The results indicate that PET and LLDPE materials can preserve the quality of this product better than PP significantly, especially in the growth of microorganisms. Adding 0.1% SB can effectively prohibit some microorganism growth and result in prolonged shelf life.

#### 3.2.3. Sensory Evaluation

Dried chili paste stored in various packaging and with/without 0.1% SB were evaluated for likeness scores of appearance, color, flavor, texture, and overall every 4 weeks of storage at room temperature (Table 5). Samples having compositions or microorganism populations outside of standards were excluded for this experiment for consumers’ safety. Overall, scores of all aspects of all samples were higher than 7.0 throughout storage up to 20 weeks, indicating the high acceptability of this product within 5 months of storage.

Normally, color is an important characteristic determining the food quality discerned by consumers at the point of sale, whereas flavor or taste components determine the overall palatability affecting the consumers’ repeated purchasing decision [29]. It was noted that there were no differences in appearance, color, and texture-liking scores of all samples during storage for 20 weeks (*p* > 0.05). This indicated that the increase in moisture content, or A_w_, as well as the increase in browning intensity of the products (Table 2 and Figure 1) during 5 months of room-temperature storage did not significantly affect the consumers’ acceptability. In contrast, the scores of flavor and overall likeness slightly decreased as storage time increased (*p* < 0.05). Moreover, these two characteristics revealed the same trend of changing in all samples. At the first 4 weeks of storage, samples without 0.1% SB had lower flavor-liking scores than samples having preservatives (*p* < 0.05), accounting for 7.19–7.39, while samples with 0.1% SB (PP+SB, PET+SB, ZL++SB) had flavor-liking scores higher than 7.5 When extending the storage, flavor-liking score was gradually decreased, which exhibited the faster rate in samples without added preservatives. This corresponded well with the higher rate of quality changes of samples without preservatives, including pH, browning index, lipid oxidation products, as well as microorganisms. Some degradation products, such as peptides or other volatiles, may reduce desirable flavors to some extent. For example, lipid oxidation products may cause undesirable compounds within the product and lead to lower flavor-liking scores. The result indicated that the flavor-liking score directly influenced overall-liking scores. Samples without added 0.1% SB exhibited lower overall-liking scores at all period times of storage, while samples having preservatives had obviously higher scores. Although both flavor and overall-liking scores of samples with 0.1% SB slightly decreased as storage time increased (*p* < 0.05), their scores were still higher than 7.0, indicating good acceptability by consumers.

#### 3.2.4. Inorganic Contaminants/Aflatoxin and Pathogens of Dried Chili Fish Paste at the 20th Week of Room-Temperature Storage (25 °C)

At the end of the 5 months of room-temperature storage, dried chili fish paste without preservatives exhibited some spoilage characteristics, i.e., excess moisture content, water activity, total microorganisms, as well as yeast and mold count. Only three samples with 0.1% SB, including PP+SB, PET+SB, and ZL+SB, had the quality characteristics in the ranges of standard [12]. To assure their safety aspects, the amount of inorganic contaminants (Pb, As, Cd, and Hg), aflatoxin, as well as pathogens, including Salmonella spp., S. aureus, B. cereus, C. perfringens, and E. coli, of those samples were determined. Table 6 reveals that those three samples with 0.1% SB have inorganic contaminants, aflatoxin, and pathogens within the limitation of the standard [12].

## 4. Conclusions

Dried chili fish paste stored in various packaging containers and with/without 0.1% SB underwent physical, chemical, as well as microbiological changes at different rates during storage at room temperature. Samples without preservatives exhibited higher rates of quality changes, including pH, browning index, lipid oxidation, microorganism count, etc. This served to limit their shelf life. The result found that PP underwent spoilage within 6 weeks, while PET and ZL had a shelf-life of 10 weeks at room temperature, as indicated by the excess of total microorganisms (TVC) as regulated by the standard. In contrast, all dried chili-paste samples treated with 0.1% SB can preserve quality for at least 20 weeks without any spoilage and still earned high consumer acceptability regardless of the packaging container. The result indicated that PET and ZL can extend the shelf life of this product longer than PP container. In addition, adding sodium benzoate at the level of 0.1% can extend the shelf life of chili paste effectively by retarding some quality changes and the microbial growth. However, even though sodium benzoate is considered safe by major regulatory agencies, there is still controversy over its effects on human health. Thus, it is necessary to be aware of the possible toxicological risks that can be caused by frequent and/or large quantities of these substances.

## Figures and Tables

**Table 1:** Chemical composition, physical properties, microbial population, and sensory evaluation of dried chili fish paste.

Parameters	Values	Parameters	Values
Physical/Chemical composition		Aflatoxin	
Moisture (%)	17.45 ± 0.24	Aflatoxin B1 (μg/kg)	ND
Protein (%)	37.63 ± 2.21	Aflatoxin B2 (μg/kg)	ND
Fat (%)	11.12 ± 2.32	Aflatoxin G1 (μg/kg)	ND
Ash (%)	15.61 ± 0.86	Aflatoxin G2 (μg/kg)	ND
Carbohydrate (%)	18.19 ± 1.84	Microbial population *	
Salt (%)	3.02 ± 0.19	Total viable count (TVC)	4.58 × 10^2^
pH	5.72 ± 0.17	Yeast and Mold count	<10
Water activity (Aw)	0.72 ± 0.06	*Salmonella* spp.	ND
Color		*S. aureus*	ND
L*	27.55 ± 0.64	*B. cereus*	<10
a*	14.49 ± 0.37	*C. perfrigens*	<10
b*	26.50 ± 0.57	*E. coli*	<10
**Inorganic contaminants**		Sensory evaluation **	
Pb (mg/kg)	ND	Appearance	7.69 ± 0.28
As (mg/kg)	ND	Color	7.01 ± 0.32
Cd (mg/kg)	<0.60	Flavor	8.23 ± 0.29
Hg (mg/kg)	0.27 ± 0.29	Texture	7.88 ± 0.41
		Overall	8.01 ± 0.24

Mean ± SD from triplicate determinations. ND, not detectable. * CFU/g sample. ** Using 50 untrained panelists.

**Table 2:** Changes in moisture content of dried chili fish paste stored in various packaging at room temperature.

Week	PP	PET	ZL	PP+SB	PET+SB	ZL + SB
2	18.68 ± 0.26 ^Ac^	18.34 ± 0.39 ^Ad^	18.15 ± 0.14 ^Be^	17.46 ± 0.11 ^Ye^*	17.75 ± 0.12 ^Yd^*	18.55 ± 0.12 ^Xd^*
4	18.72 ± 0.40 ^Ac^	19.04 ± 0.32 ^Ac^	18.16 ± 0.09 ^Be^	17.88 ± 0.10 ^Yd^*	17.88 ± 0.33 ^Yd^*	19.02 ± 0.10 ^Xc^*
6	18.77 ± 0.09 ^Bc^	19.60 ± 0.18 ^Ac^	18.77 ± 0.32 ^Bd^	17.93 ± 0.26 ^Yd^*	18.14 ± 0.09 ^Yc^*	18.92 ± 0.24 ^Xc^
8	18.69 ± 0.11 ^Bc^	19.79 ± 0.15 ^Ac^	18.92 ± 0.29 ^Bd^	18.35 ± 0.33 ^Yc^*	18.10 ± 0.10 ^Yc^*	19.06 ± 0.12 ^Xc^
10	19.24 ± 0.23 ^Bb^	19.65 ± 0.22 ^Ac^	18.78 ± 0.21 ^Cd^	18.22 ± 0.21 ^Yc^*	18.22 ± 0.14 ^Yc^*	19.44 ± 0.17 ^Xb^*
12	19.30 ± 0.25 ^Bb^	20.03 ± 0.30 ^Ab^	19.15 ± 0.17 ^Bc^	18.32 ± 0.09 ^Zc^*	18.76 ± 0.12 ^Yb^*	19.35 ± 0.09 ^Xb^
14	20.09 ± 0.14 ^A^^a^	20.11 ± 0.23 ^Ab^	19.23 ± 0.07 ^Bc^	18.99 ± 0.14 ^Yb^*	18.67 ± 0.28 ^Yb^*	19.62 ± 0.06 ^Xab^*
16	20.14 ± 0.30 ^Aa^	20.15 ± 0.09 ^Ab^	19.42 ± 0.09 ^Bb^	19.07 ± 0.14 ^Yb^*	18.69 ± 0.30 ^Zb^*	19.55 ± 0.15 ^Xb^
18	20.02 ± 0.15 ^Ba^	20.54 ± 0.16 ^A^^a^	19.68 ± 0.17 ^Cb^	19.69 ± 0.29 ^Xa^*	19.12 ± 0.24 ^Ya^*	19.56 ± 0.28 ^Xb^
20	20.01 ± 0.11 ^Ba^	20.65 ± 0.22 ^Aa^	20.02 ± 0.23 ^Ba^	19.78 ± 0.10 ^Xa^*	19.25 ± 0.16 ^Ya^*	19.87 ± 0.15 ^Xa^*

Mean ± SD from triplicate determinations. Different uppercase letters in the same row indicate significant differences (*p* < 0.05) due to packaging material at the same period of storage time (without 0.1% SB: A,B,C ; with 0.1% SB: X,Y,Z). Significant differences between with/without added 0.1% SB of samples stored in the same packaging material are indicated by an asterisk (*) on the added preservative samples (*p* < 0.05). Different lowercase letters in the same column denote significant differences (*p* < 0.05) due to storage time.

**Table 3:** Changes in color of dried chili fish paste stored in various packaging at room temperature.

Week	PP	PET	ZL	PP+SB	PET+SB	ZL+SB
	L*					
2	26.02 ± 0.27 ^Ba^	28.42 ± 0.18 ^Aa^	25.92 ± 0.30 ^Ba^	26.42 ± 0.44 ^Ya^	27.86 ± 0.41 ^Xa^	27.23 ± 0.22 ^Xa^*
4	24.34 ± 0.65 ^Bb^	27.97 ± 0.44 ^Aab^	25.88 ± 0.22 ^Ba^	25.47 ± 0.18 ^Yb^*	28.01 ± 0.34 ^Xa^	27.65 ± 0.51 ^Xa^*
6	24.88 ± 0.06 ^Bb^	26.63 ± 0.29 ^Ab^	26.06 ± 0.69 ^Aa^	25.39 ± 0.23 ^Yb^*	26.96 ± 0.30 ^Xb^	25.31 ± 0.25 ^Yb^*
8	23.91 ± 0.51 ^Bb^	26.61 ± 0.20 ^Ab^	24.21 ± 0.15 ^Bb^	25.26 ± 0.51 ^Zb^*	26.88 ± 0.40 ^Xb^	25.98 ± 0.09 ^Yb^*
10	22.06 ± 0.33 ^Cc^	25.52 ± 0.16 ^Ac^	24.67 ± 0.20 ^Bb^	25.64 ± 0.26 ^Yb^*	26.01 ± 0.27 ^Xc^*	25.66 ± 0.36 ^Yb^*
12	22.48 ± 0.20 ^Cc^	25.66 ± 0.32 ^Ac^	24.35 ± 0.19 ^Bb^	24.05 ± 0.29 ^Yc^*	26.41 ± 0.32 ^Xbc^*	24.86 ± 0.22 ^Yc^*
14	22.15 ± 0.19 ^Cc^	26.02 ± 0.33 ^Abc^	23.92 ± 0.41 ^Bc^	24.12 ± 0.42 ^Zc^*	25.89 ± 0.29 ^Xd^*	24.92 ± 0.23 ^Yc^*
16	20.66 ± 0.11 ^Bd^	24.97 ± 0.42 ^Ad^	24.01 ± 0.33 ^Abc^	23.99 ± 0.42 ^Yc^*	26.21 ± 0.55 ^Xbc^*	23.05 ± 0.60 ^Yd^*
18	19.59 ± 0.25 ^Ce^	24.00 ± 0.19 ^Ae^	23.25 ± 0.30 ^Bc^	23.65 ± 0.33 ^Yc^*	25.13 ± 0.42 ^Xe^*	23.21 ± 0.13 ^Yd^
20	19.63 ± 0.42 ^Ce^	24.08 ± 0.33 ^Ae^	22.96 ± 0.25 ^Bd^	23.81 ± 0.49 ^Yc^*	25.22 ± 0.31 ^Xe^*	23.26 ± 0.44 ^Yd^*
	a*					
2	14.61 ± 0.32 ^Ba^	14.20 ± 0.41 ^Ba^	16.02 ± 0.23 ^Aa^	15.33 ± 0.23 ^Xa^*	15.24 ± 0.26 ^Xa^*	14.79 ± 0.32 ^Ya^*
4	14.28 ± 0.11 ^Bb^	13.60 ± 0.16 ^Cab^	15.31 ± 0.16 ^Ab^	15.20 ± 0.33 ^Xa^*	15.20 ± 0.24 ^Xa^*	14.65 ± 0.22 ^Ya^*
6	14.99 ± 0.26 ^Ba^	13.23 ± 0.21 ^Cb^	15.88 ± 0.45 ^Aa^	14.27 ± 0.18 ^Ybc^*	15.03 ± 0.30 ^Xa^*	14.39 ± 0.11 ^Yb^*
8	13.02 ± 0.12 ^Cd^	14.39 ± 0.41 ^Ba^	15.92 ± 0.22 ^Aa^	14.55 ± 0.29 ^Yb^*	15.11 ± 0.23 ^Xa^*	14.27 ± 0.08 ^Yb^*
10	13.25 ± 0.49 ^Cd^	14.09 ± 0.30 ^Ba^	15.43 ± 0.22 ^Ab^	14.49 ± 0.25 ^Yb^*	14.99 ± 0.20 ^Xa^*	14.20 ± 0.35 ^Yb^*
12	13.98 ± 0.25 ^Bb^	14.11 ± 0.22 ^Ba^	15.44 ± 0.16 ^Ab^	14.02 ± 0.22 ^Zc^	14.25 ± 0.15 ^Yb^	14.89 ± 0.18 ^Xa^*
14	13.92 ± 0.22 ^Bb^	14.54 ± 0.20 ^Aa^	14.97 ± 0.20 ^Ac^	14.11 ± 0.14 ^Yc^	14.22 ± 0.22 ^Yb^*	14.76 ± 0.19 ^Xa^*
16	14.01 ± 0.19 ^Bb^	13.18 ± 0.33 ^Cb^	15.16 ± 0.33 ^Abc^	13.99 ± 0.33 ^Zc^	14.53 ± 0.14 ^Yab^*	14.72 ± 0.14 ^Xa^*
18	13.68 ± 0.20 ^Bc^	14.15 ± 0.34 ^Ba^	15.22 ± 0.52 ^Abc^	13.97 ± 0.07 ^Yc^*	14.66 ± 0.31 ^Xab^*	14.88 ± 0.22 ^Xa^*
20	14.88 ± 0.44 ^Ba^	13.88 ± 0.25 ^Cab^	15.28 ± 0.15 ^Abc^	14.25 ± 0.32 ^Ybc^*	15.05 ± 0.21 ^Xa^*	14.49 ± 0.39 ^Yab^*
	b*					
2	26.22 ± 0.10 ^e^	26.31 ± 0.20 ^d^	26.07 ± 0.22 ^c^	26.15 ± 0.14 ^Xd^	26.00 ± 0.25 ^Xd^*	25.43 ± 0.20 ^Ye^*
4	26.25 ± 0.20 ^Ae^	26.55 ± 0.18 ^Ad^	25.90 ± 0.18 ^Bc^	26.40 ± 0.22 ^Xcd^	25.95 ± 0.22 ^Yd^*	26.22 ± 0.19 ^Xcd^*
6	26.07 ± 0.18 ^Be^	26.70 ± 0.16 ^Ad^	26.55 ± 0.19 ^Ab^	26.22 ± 0.07 ^Xd^	26.04 ± 0.32 ^Yd^*	26.09 ± 0.13 ^Yd^*
8	26.90 ± 0.33 ^Bd^	28.24 ± 0.09 ^Ac^	26.56 ± 0.24 ^Bb^	26.42 ± 0.13 ^Ycd^*	27.55 ± 0.40 ^Xbc^*	26.65 ± 0.16 ^Yc^
10	26.82 ± 0.24 ^Bd^	28.09 ± 0.22 ^Ac^	26.49 ± 0.22 ^Bb^	26.20 ± 0.15 ^Zd^*	27.30 ± 0.09 ^Xc^*	26.61 ± 0.24 ^Yc^
12	27.14 ± 0.36 ^Bc^	28.99 ± 0.20 ^Ab^	27.22 ± 0.15 ^Ba^	26.88 ± 0.20 ^Ybc^*	28.01 ± 0.17 ^Xb^*	27.88 ± 0.32 ^Xab^*
14	27.34 ± 0.18 ^Bc^	29.11 ± 0.30 ^Ab^	27.03 ± 0.16 ^Ba^	26.75 ± 0.31 ^Ybc^*	27.97 ± 0.29 ^Xb^*	27.81 ± 0.22 ^Xab^*
16	27.99 ± 0.29 ^Bb^	29.40 ± 0.33 ^Aa^	27.00 ± 0.21 ^Ba^	27.04 ± 0.44 ^Zb^*	28.04 ± 0.26 ^Xb^*	27.59 ± 0.09 ^Yb^*
18	30.15 ± 0.45 ^Aa^	29.41 ± 0.24 ^Ba^	27.20 ± 0.39 ^Ca^	27.04 ± 0.29 ^Zb^*	28.65 ± 0.12 ^Xa^*	28.02 ± 0.31 ^Ya^*
20	30.77 ± 0.29 ^Aa^	29.28 ± 0.17 ^Ba^	27.15 ± 0.26 ^Ca^	28.11 ± 0.13 ^Ya^*	28.70 ± 0.09 ^Xa^*	27.89 ± 0.20 ^Zab^*

Mean ± SD from triplicate determinations. Different uppercase letters in the same row indicate significant differences (*p* < 0.05) due to packaging material at the same period of storage time (without 0.1% SB: A,B,C; with 0.1% SB: X,Y,Z). Significant differences between with/without added 0.1% SB samples stored in the same packaging material are indicated by an asterisk (*) on the added preservative samples (*p* < 0.05). Different lowercase letters in the same column denote significant differences (*p* < 0.05) due to storage time.

**Table 4:** Changes in total viable count (TVC) and yeast and mold of dried chili fish paste during storage at room temperature.

Week	Total Viable Count (TVC) *					
	PP	PET	ZL	PP+SB	PET+SB	ZL+SB
2	2.08 × 10^3^	9.25 × 10^2^	7.11 × 10^2^	2.87 × 10^2^	3.12 × 10^2^	7.02 × 10^2^
4	5.22 × 10^3^	2.09 × 10^3^	9.97 × 10^2^	3.03 × 10^2^	5.69 × 10^2^	9.01 × 10^2^
6	1.05 × 10^4^	8.34 × 10^3^	5.42 × 10^3^	6.09 × 10^2^	9.09 × 10^2^	9.59 × 10^2^
8	5.23 × 10^4^	7.99 × 10^3^	9.34 × 10^3^	6.55 × 10^2^	9.26 × 10^2^	1.22 × 10^3^
10	-	2.98 × 10^4^	1.22 × 10^4^	9.02 × 10^2^	1.08 × 10^3^	1.18 × 10^3^
12	-	-	-	1.11 × 10^3^	1.25 × 10^3^	3.35 × 10^3^
14	-	-	-	2.63 × 10^3^	5.18 × 10^3^	7.44 × 10^3^
16	-	-	-	6.06 × 10^3^	5.99 × 10^3^	7.29 × 10^3^
18	-	-	-	6.03 × 10^3^	8.01 × 10^3^	8.67 × 10^3^
20	-	-	-	6.41 × 10^3^	8.29 × 10^3^	8.60 × 10^3^
	Yeast and Mold count *					
2	35	22	33	<10	<10	<10
4	78	45	50	<10	<10	18
6	106	87	52	22	<10	15
8	130	88	77	44	12	20
10	-	90	82	50	30	42
12	-	102	88	78	65	56
14	-	-	115	74	71	63
16	-	-	-	80	77	79
18	-	-	-	78	78	75
20	-	-	-	82	80	77

* CFU/g sample.

**Table 5:** Sensory evaluation of dried chili fish paste during storage in various packaging at room temperature.

Week of Storage	Appearance					
	PP	PET	ZL	PP+SB	PET+SB	ZL+SB
4	7.28 ± 0.31 ^B^	7.43 ± 0.20 ^B^	7.62 ± 0.15 ^A^	7.62 ± 0.22 *	7.60 ± 0.22	7.73 ± 0.18
8	-	7.39 ± 0.15 ^B^	7.58 ± 0.16 ^A^	7.60 ± 0.13	7.61 ± 0.13 *	7.67 ± 0.13
12	-	-	-	7.66 ± 0.14	7.52 ± 0.18	7.64 ± 0.16
16	-	-	-	7.55 ± 0.20	7.54 ± 0.20	7.50 ± 0.12
20	-	-	-	7.60 ± 0.31	7.63 ± 0.31	7.62 ± 0.36
	Color					
4	7.01 ± 0.19 ^B^	7.21 ± 0.16 ^A^	7.26 ± 0.09 ^A^	7.25 ± 0.29	7.20 ± 0.17	7.01. ± 0.23
8	-	7.26 ± 0.22	7.33 ± 0.25	7.15 ± 0.22	7.20 ± 0.27	7.04 ± 0.24
12	-	-	-	7.29 ± 0.12	7.25 ± 0.19	7.19 ± 0.10
16	-	-	-	7.01 ± 0.29	7.24 ± 0.14	7.06 ± 0.22
20	-	-	-	7.17 ± 0.21	7.01 ± 0.06	7.12 ± 0.10
	Flavor					
4	7.19 ± 0.32	7.35 ± 0.23 ^a^	7.39 ± 0.33 ^a^	7.78 ± 0.17 ^a^*	7.82 ± 0.10 ^a^*	7.78 ± 0.17 ^a^*
8	-	6.92 ± 0.26 ^b^	7.06 ± 0.24 ^b^	7.42 ± 0.20 ^Bb^	7.86 ± 0.29 ^Aa^*	7.82 ± 0.20 ^Aa^*
12	-	-	-	7.41 ± 0.16 ^Bb^	7.52 ± 0.22 ^Bb^	7.81 ± 0.16 ^Aa^
16	-	-	-	7.18 ± 0.18 ^Cc^	7.43 ± 0.16 ^Bb^	7.63 ± 0.18 ^Ab^
20	-	-	-	7.22 ± 0.23 ^Bc^	7.39 ± 0.19 ^Bb^	7.65 ± 0.23 ^Ab^
	Texture					
4	7.82 ± 0.19	7.79 ± 0.20	7.84 ± 0.17	7.73 ± 0.16	7.80 ± 0.13	7.78 ± 0.16
8	-	7.83 ± 0.19	7.79 ± 0.23	7.77 ± 0.14	7.74 ± 0.12	7.80 ± 0.14
12	-	-	-	7.65 ± 0.29	7.70 ± 0.22	7.65 ± 0.20
16	-	-	-	7.60 ± 0.22	7.75 ± 0.15	7.67 ± 0.19
20	-	-	-	7.62 ± 0.26	7.70 ± 0.16	7.60 ± 0.22
	Overall					
4	7.34 ± 0.22	7.29 ± 0.11 ^a^	7.01 ± 0.09 ^a^	7.92 ± 0.22 ^Aa^*	7.83 ± 0.14 ^AB^*	7.72 ± 0.16 ^B^*
8	-	7.03 ± 0.27 ^Ab^	6.78 ± 0.21 ^Bb^	7.79 ± 0.15 ^a^	7.84 ± 0.13 *	7.80 ± 0.13 *
12	-	-	-	7.80 ± 0.13 ^a^	7.70 ± 0.20	7.65 ± 0.29
16	-	-	-	7.82 ± 0.21 ^a^	7.62 ± 0.27	7.60 ± 0.21
20	-	-	-	7.39 ± 0.30 ^Bb^	7.65 ± 0.17 ^A^	7.71 ± 0.29 ^A^

Mean ± SD from triplicate determinations. Different uppercase letters in the same row indicate significant differences (*p* < 0.05) due to packaging material at the same period of storage time (without 0.1% SB: A,B,C). Significant differences between with/without added 0.1% SB in samples stored in the same packaging material are indicated by an asterisk (*) on the added preservative samples.

**Table 6:** Inorganic contaminants, aflatoxin, and pathogens in dried chili fish paste stored in the chosen packaging at the 20th week of room-temperature storage.

Parameters	PP+SB	PET+SB	ZL+SB
**Inorganic contaminants**			
Pb (mg/kg)	ND	ND	ND
As (mg/kg)	ND	ND	ND
Cd (mg/kg)	<0.45	<1.12	<0.66
Hg (mg/kg)	0.18 ± 0.02	0.29 ± 0.19	0.32 ± 0.14
**Aflatoxin**			
Aflatoxin B1 (μg/kg)	ND	ND	ND
Aflatoxin B2 (μg/kg)	ND	ND	ND
Aflatoxin G1 (μg/kg)	ND	ND	ND
Aflatoxin G2 (μg/kg)	ND	ND	ND
**Pathogen**			
*Salmonella* spp.	ND	ND	ND
*S* *. aureus*	ND	ND	ND
*B* *. cereus*	<10	12	<10
*C* *. perfringens*	ND	15	20
*E* *. coli*	<10	<10	<10

Mean ± SD from triplicate determinations. ND, not detectable.
